# Clinical outcomes in spontaneous subarachnoid hemorrhage after introduction of continuous intra-arterial vasospasmolysis for treatment of refractory delayed cerebral ischemia

**DOI:** 10.3389/fneur.2025.1636083

**Published:** 2025-12-03

**Authors:** Axel Neulen, Verena Fassl, Elena Kurz, Andreas Kramer, Annekathrin Silvia Nedwed, Marc A. Brockmann, Florian Ringel, Carolin Brockmann

**Affiliations:** 1 Department of Neurosurgery, University Medical Center of the Johannes Gutenberg-University of Mainz, Mainz, Germany; 2 Department of Neurosurgery, LMU University Hospital, LMU Munich, Munich, Germany; 3 Institute of Medical Biostatistics, Epidemiology and Informatics, University Medical Center of the Johannes Gutenberg-University of Mainz, Mainz, Germany; 4 Department of Neuroradiology, University Medical Center of the Johannes Gutenberg-University of Mainz, Mainz, Germany

**Keywords:** subarachnoid hemorrhage, cerebral perfusion, delayed cerebral ischemia, nimodipine, vasospasm, endovascular

## Abstract

**Background:**

In patients with refractory delayed cerebral ischemia (DCI) after spontaneous subarachnoid hemorrhage (SAH), endovascular therapy of cerebral vasospasms is a treatment option. In our center, continuous intra-arterial vasospasmolysis with nimodipine (ciaN) has been introduced as the standard endovascular therapy for cerebral vasospasms since 2016. This study investigated the outcomes of SAH patients before and after introduction of ciaN.

**Methods:**

Data pertaining to all patients treated for SAH in our center between 2011 and 2021 were retrospectively recorded.

**Results:**

145 patients before (pre-ciaN group) and 147 after (ciaN group) introduction of ciaN met the inclusion criteria. 36 patients in the pre-ciaN group and 51 in the ciaN group received endovascular vasospasm treatment. At discharge, outcomes tended to improve in the ciaN group. After 6 months, there was a significantly improved outcome in the ciaN group (mRS 0–2, Fisher’s exact test). After propensity score matching, there were no significant differences between the pre-ciaN and ciaN groups in the subgroups of patients without endovascular vasospasm treatments. Conversely, in the subgroups of patients who had received endovascular vasospasm treatments, there was a significantly improved outcome at discharge and after 6 months, and a significant reduction of DCI-associated infarctions.

**Conclusion:**

Outcome after spontaneous subarachnoid hemorrhage has improved since the introduction of ciaN in our center. Our data indicate a contribution of the changes in treatment standard for endovascular vasospasm therapies from angioplasties to ciaN. Prospective studies are needed to compare the effect of ciaN in DCI with standard medical therapy.

## Introduction

Spontaneous subarachnoid hemorrhage (SAH), which is caused by rupture of an intracranial aneurysm in most cases, is a type of hemorrhagic stroke (1, 2). It accounts for approximately 5% of all strokes in most western countries (3). Compared with other forms of stroke, patients with SAH are younger with a mean age of approximately 55 years, as reported in most studies (4). Brain injury after SAH develops in two phases: Early brain injury occurs as the direct consequence of bleeding and the associated transient global cerebral ischemia (5, 6). In contrast, delayed cerebral ischemia (DCI) occurs within the first weeks after SAH onset (5). The pathophysiology of DCI is complex. In addition to vasospasms of large intracranial arteries, microvasospasms, microthrombosis, cortical spreading depressions, and inflammation have been described as contributing factors (5) It leads to cerebral hypoperfusion and, in some cases, to cerebral infarctions (DCI-associated infarctions) and is a central factor influencing neurological outcomes (5).

Oral nimodipine in the first weeks after SAH is the only evidence-based therapy that reduces the rates of delayed infarctions and improves outcomes (7). If DCI still occurs, current guidelines recommend inducing hypertension (8), despite a lack of prospective studies showing its effects on outcomes. In case of DCI that does not respond to induced hypertension, endovascular therapies targeting cerebral vasospasms can be applied (8). These endovascular approaches aim to improve brain perfusion by mitigating vasospasms either by angioplasty of large vasospastic arteries or by intra-arterial infusion of vasodilating drugs into vasospastic arteries.

Intra-arterial infusion of vasodilating drugs can be performed either as “single-shot” application—that is, the intra-arterial infusion is terminated after angiography—or as continuous intra-arterial infusion (9–14). For continuous intra-arterial infusion, a catheter is commonly left behind in an internal carotid or vertebral artery, depending on the location of the vasospasms. To prevent embolism from the intra-arterial catheters, anticoagulation or antiplatelet therapy is necessary until the catheter is removed. Previous studies have found that continuous intra-arterial infusion into vessels affected by vasospasms is a secure method that significantly reduces cerebral angiographic vasospasms and leads to a low complication rate (9–14). However, it remains unclear whether the intra-arterial infusion of nimodipine in case of refractory DCI in patients with SAH improves outcomes.

At our center, the endovascular treatment of vasospasms is offered to patients with SAH and refractory DCI as rescue treatment. Until 2015, endovascular treatment comprised a single-shot intra-arterial infusion of nimodipine and/or angioplasty of vasospastic arteries. In 2016, the standard was changed to single-shot intra-arterial infusion of nimodipine or continuous intra-arterial infusion of nimodipine (ciaN). Angioplasty was no longer routinely performed because we considered ciaN to be similarly effective and associated with less complications. This study aimed to analyze the effects of the change in the treatment standard by comparing the outcomes before and after the switch in the method for endovascular vasospasm treatment in two cohorts of patients with SAH.

## Methods

### Ethics approval

The study was approved by the local Ethics Committee (Ethikkommission der Landesärztekammer Rheinland Pfalz). Because of the retrospective nature of the study, the requirement for informed consent was waived.

### Patients and study design

We retrospectively identified all patients aged ≥18 years, treated at the neurosurgical intensive care unit of the Medical Center of the Johannes Gutenberg-University of Mainz, Germany, with the diagnosis of spontaneous SAH between January 01, 2011, and June 30, 2021. The patients were assigned to the cohorts before (January 01, 2011, to December 31, 2015) and after (July 01, 2016, to June 30, 2021) the introduction of ciaN, which replaced angioplasty as the standard endovascular therapy for refractory DCI. During the period from January 2016 to June 2016, both methods (ciaN and angioplasty) were used. Therefore, patients treated during this period were excluded.

Furthermore, patients with available imaging data (either cranial computed tomography or cranial magnetic resonance imaging) acquired between 14 and 28 days after SAH onset to be analyzed for DCI-associated infarctions and available clinical outcome data at hospital discharge were included.

### Data analysis and study endpoints

Data were collected in anonymized data tables.

Data on the patients (age, sex, smoking, and arterial hypertension), bleeding events (presence of an aneurysm as bleeding source, Hunt and Hess [H&H] and Fisher scores), and whether an endovascular treatment of refractory DCI was performed were extracted from the patient charts. Data on the clinical outcomes (favorable and unfavorable outcomes) were extracted from the patient charts. A favorable outcome was defined as an mRS score of 0–2; an unfavorable outcome was defined as an mRS score of 3–6. The outcomes were evaluated at hospital discharge and at clinical follow-up after 6 months. In cases of missing data 6 months after SAH, outcome data at 3–12 months after SAH were evaluated, if available.

As an additional outcome parameter, we analyzed the presence of DCI-associated infarctions. To analyze DCI-associated infarctions, imaging data (cranial computed tomography [CT] or magnetic resonance imaging [MRI]) acquired 14–28 days after SAH onset were analyzed by a neuroradiologist blinded to the other data. In case of the presence of cerebral infarctions, the complete history of cranial imaging starting from SAH onset was analyzed, and infarctions were identified as DCI-related if no other causes were present.

### Clinical management of SAH and DCI

Clinical management was performed according to our institutional protocols as described (9, 15–19). In brief, SAH was diagnosed using cranial CT scans. CT angiography (CTA) and digital subtraction angiography were performed to identify the source of the bleeding. If an aneurysm was present, the case was discussed between the endovascular and surgical specialists, and the aneurysm was treated after interdisciplinary consensus by either surgical clipping or endovascular coiling within 24 h. If necessary, external ventricular or lumbar drains were used to treat hydrocephalus. Patients were closely monitored neurologically. All patients were admitted to the intensive care. After securing the bleeding source and if deemed appropriate by the attending neurointensivists, some patients were transferred to the intermediate care unit and, in some cases, to the neurosurgical ward. Here, too, the patients were closely monitored neurologically. In unconscious patients, if indicated, intraparenchymal probes placed in the frontal region were used for ICP and ptiO_2_ (brain tissue oxygenation) monitoring.

All patients received oral nimodipine at a dose of 60 mg every 4 h for 3 weeks after SAH onset. DCI was suspected in cases of global or focal neurological deterioration or, in unconscious patients, in cases of increasing Doppler sonographic flow velocities of the intracranial arteries or a decrease in ptiO_2_ to <15 mmHg. In these cases, hypertension was induced, and CT perfusion imaging and CTA were performed. In cases with refractory DCI that are not responsive to induced hypertension, cerebral hypoperfusion, and severe angiographic vasospasms, digital subtraction angiography and endovascular vasospasm therapies were indicated after interdisciplinary consensus between neurointensivists and endovascular specialists (9).

In addition to the switch from angioplasties to ciaN, routine administration of magnesium and simvastatin was discontinued during the study period following the publication of the MASH-2 trial (20) and the STASH trial (21). Otherwise, the relevant treatment standards remained constant throughout the observation period.

### Endovascular vasospasm therapy

#### ciaN

For ciaN, the common femoral artery was routinely used to obtain subpetrous vascular access using a 6-Fr guiding catheter. Furthermore, 1–2 microcatheters were placed in the extracranial internal carotid artery (ICA) or vertebral artery (VA) according to the site of impending infarction. Nimodipine (Nimotop® S, 10 mg/50 mL, Bayer Vital GmbH GB Pharma) was initially administered at a rate of 2 mg/h (10 mL/h) in combination with 10 mL/h of heparin (Ratiopharm, Ulm, Germany), 1 IU/mL resulting in an infusion rate of 20 mL/h to prevent catheter obstruction or thrombus apposition. Depending on the clinical and radiographic course of the individual patient, the nimodipine administration rate was then reduced successively. Platelet inhibition with Tirofiban (Advanz Pharma Corp., London, United Kingdom) at a body weight-adapted dose was performed to prevent thromboembolic complications.

#### Single-shot intra-arterial vasospasmolysis with nimodipine

Single-shot intra-arterial vasospasmolysis with nimodipine was performed using the same approach used for ciaN. One milligram of nimodipine was administered through the guiding catheter positioned in either the ICA or VA for 30 min.

#### Angioplasty

Using a 6-Fr guiding catheter in the desired ICA or VA extracranially, a microcatheter was advanced over a 0.014-inch microwire. Noncompliant balloons were selected based on vessel diameter and were positioned at the desired stenosis to perform PTA.

### Statistical analysis

The aim of the statistical analysis was to determine whether the change in the standard therapy for refractory DCI had an influence on the outcomes (DCI-associated infarctions and favorable outcome at discharge and at 6 months). For this purpose, contingency tables were created, and Fisher’s exact tests were performed.^1^ Two- sided *p*-values <0.05 were used to denote statistical significance.

Propensity score matching adjusting for the categories age, sex, H&H and Fisher scores, hypertension, active nicotine use, and detection of an aneurysm as bleeding source was used to isolate the treatment effect. We estimated inverse probability weights (using the R package WeightIt, v. 1.4.0) and created an integer-weighted pseudo-population by normalizing weights to mean 1 and replicating rows accordingly. Counts shown in consistency tables therefore reflect the weighted pseudo-sample and do not equal raw patient counts. The effective sample size (ESS) of 136 was reported to contextualize precision.

## Results

### Patients

**Figure 1:**
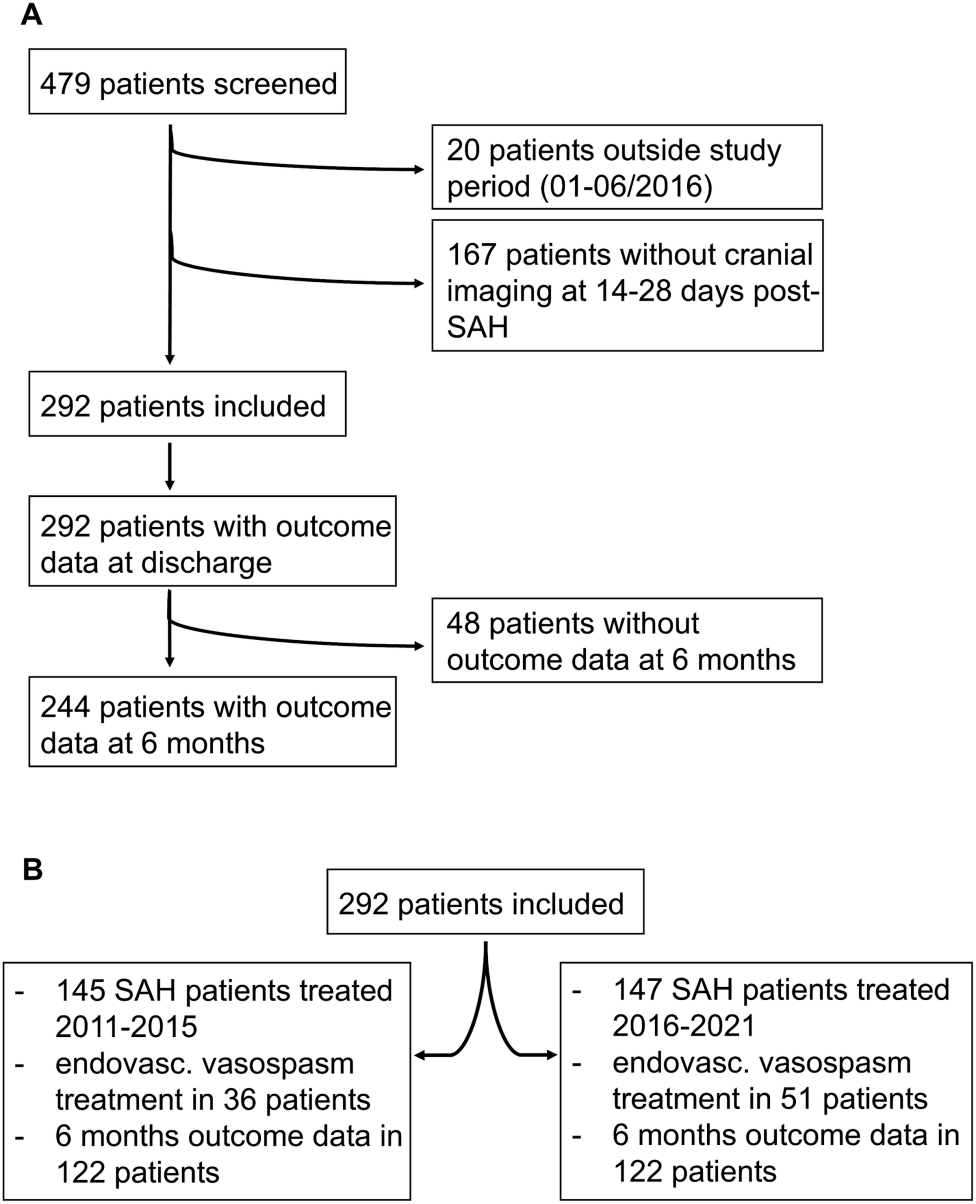
Patients. **(A)** Identification and exclusion of patients included in the evaluation. **(B)** Shows patients included in the cohorts before and after the introduction of ciaN.

A total of 479 consecutive patients treated at our department between January 01, 2011, and June 30, 2021, with the diagnosis of spontaneous SAH were screened. Of them, 20 were treated between January 01, 2016, and June 30, 2016 and were thus excluded. Of the remaining 459 patients, clinical outcome data and cranial CT or MRI acquired 14–28 days after SAH onset were available for 292 patients, who were included in our study. Of the 292 patients, 145 were treated before the introduction of ciaN (2011–2015), and 147 were treated after the introduction of ciaN (2016–2021). Figure 1 presents a detailed overview of the included patients.

**Figure 2:**
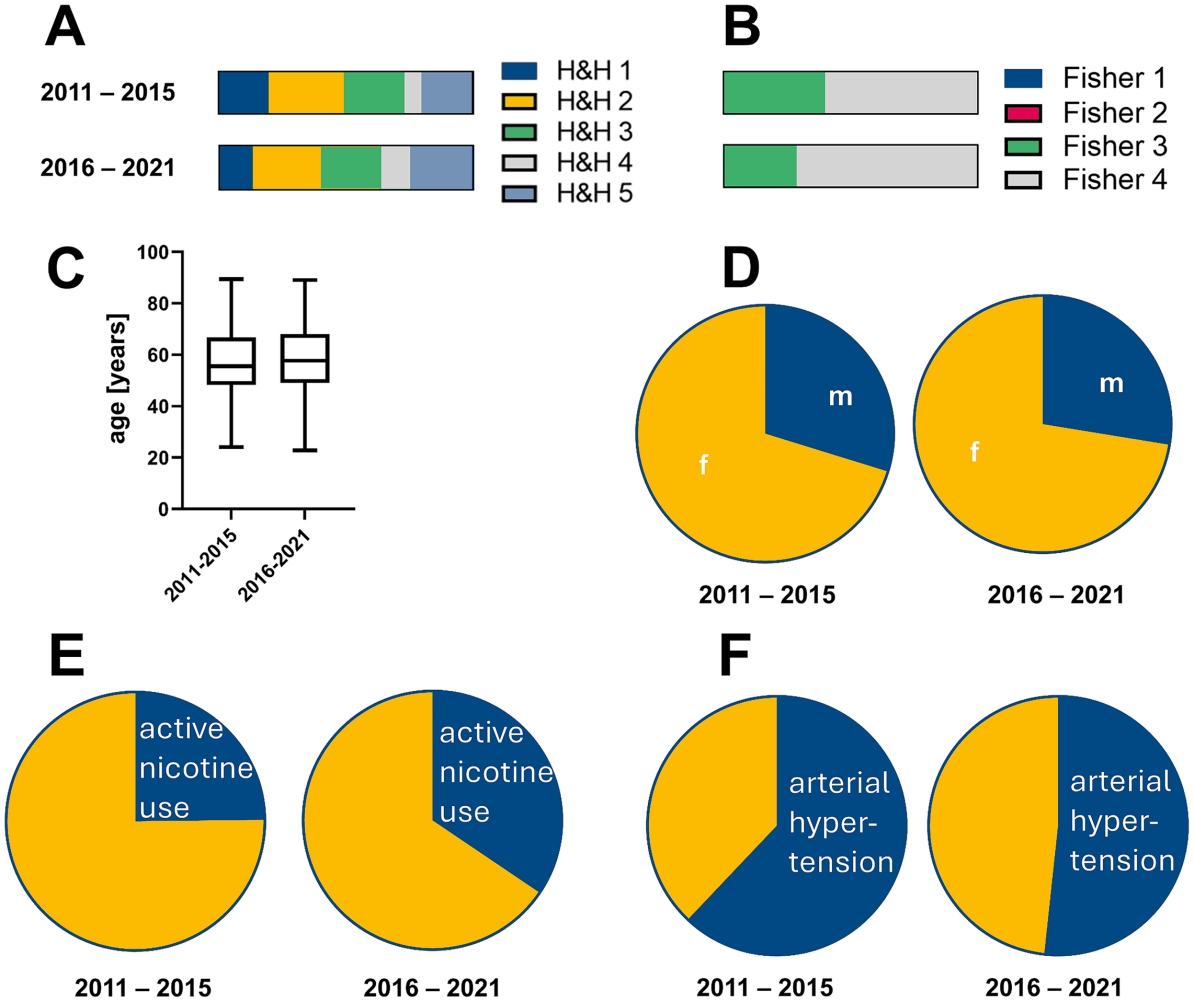
Patient characteristics. **(A–F)** Show the characteristics of the patients included in the cohorts before and after the introduction of ciaN.

An aneurysm as bleeding source was detected in 92% (pre-ciaN group) and 91% (ciaN group). No significant differences in patient sex, age were observed between the two groups. Regarding the H&H scores and Fisher scores at hospital admission, the proportion of patients with H&H scores of 4 and 5 and Fisher 4 were higher in the ciaN group than in the pre-ciaN group. Further heterogeneities were observed concerning arterial hypertension and active nicotine use. Figure 2 presents detailed data on the patients and the severity of the bleeding event.

### Endovascular interventions for treating severe vasospasms

Of the 292 patients included in our study, endovascular vasospasm treatment for refractory DCI was performed in 87 patients (29.8%): 36 patients in the pre-ciaN group (24.8%) and 51 patients in the ciaN group (34.7%).

The treatments performed in the 36 patients in the pre-ciaN group were balloon angioplasty of the vasospastic cerebral arteries in 19 patients, together with intra-arterial single-shot application of nimodipine. Only one patient received a continuous intra-arterial nimodipine infusion. The other patients received intra-arterial single-shot applications of nimodipine only. Of the 36 patients, 27 underwent more than one endovascular intervention.

Of the 51 patients in the ciaN cohort, only three underwent balloon angioplasty of the vasospastic cerebral arteries. In total, 21 patients received continuous intra-arterial nimodipine infusions. The remaining 27 patients received single-shot applications of nimodipine only. Thirty-seven patients underwent more than one endovascular intervention.

Of the patients admitted with high H&H scores, a similar proportion received endovascular vasospasm treatments in the ciaN and pre-ciaN groups, whereas, in patients with low H&H scores, the rate of endovascular treatments tended to be higher in the ciaN group: Of the 92 patients admitted with H&H scores of 4 or 5, 31 (33.7%) obtained endovascular vasospasm treatments (pre-ciaN cohort: 30.8% vs. ciaN cohort: 35.8%, *n.s.*) compared with 56 (28.0%) of the 200 patients admitted with H&H scores of 1–3 (pre-ciaN cohort: 22.6% vs. ciaN cohort: 34.0%, *n.s.*).

The average duration of ciaN was 5 days, up to 15 days in 1 case. Regarding ciaN, there were some minor complications. Microcatheter obstruction occurred in some cases, resulting in pressure alarms from the infusion pumps. Dislocation of a microcatheter also occurred. In these cases, the affected microcatheter was removed or replaced if necessary. Furthermore, a vessel dissection at the arterial access sheath occurred in two cases, which did not require specific treatment. In one case, follow-up imaging showed cerebral hyperperfusion, which resolved after ciaN was discontinued. However, no complications of ciaN affecting long term outcome were detected. ICP elevations or relevant cerebral edema associated with intraarterial Nimodipine infusion or infections induced by ciaN were not observed.

### DCI-associated cerebral infarctions

**Figure 3:**
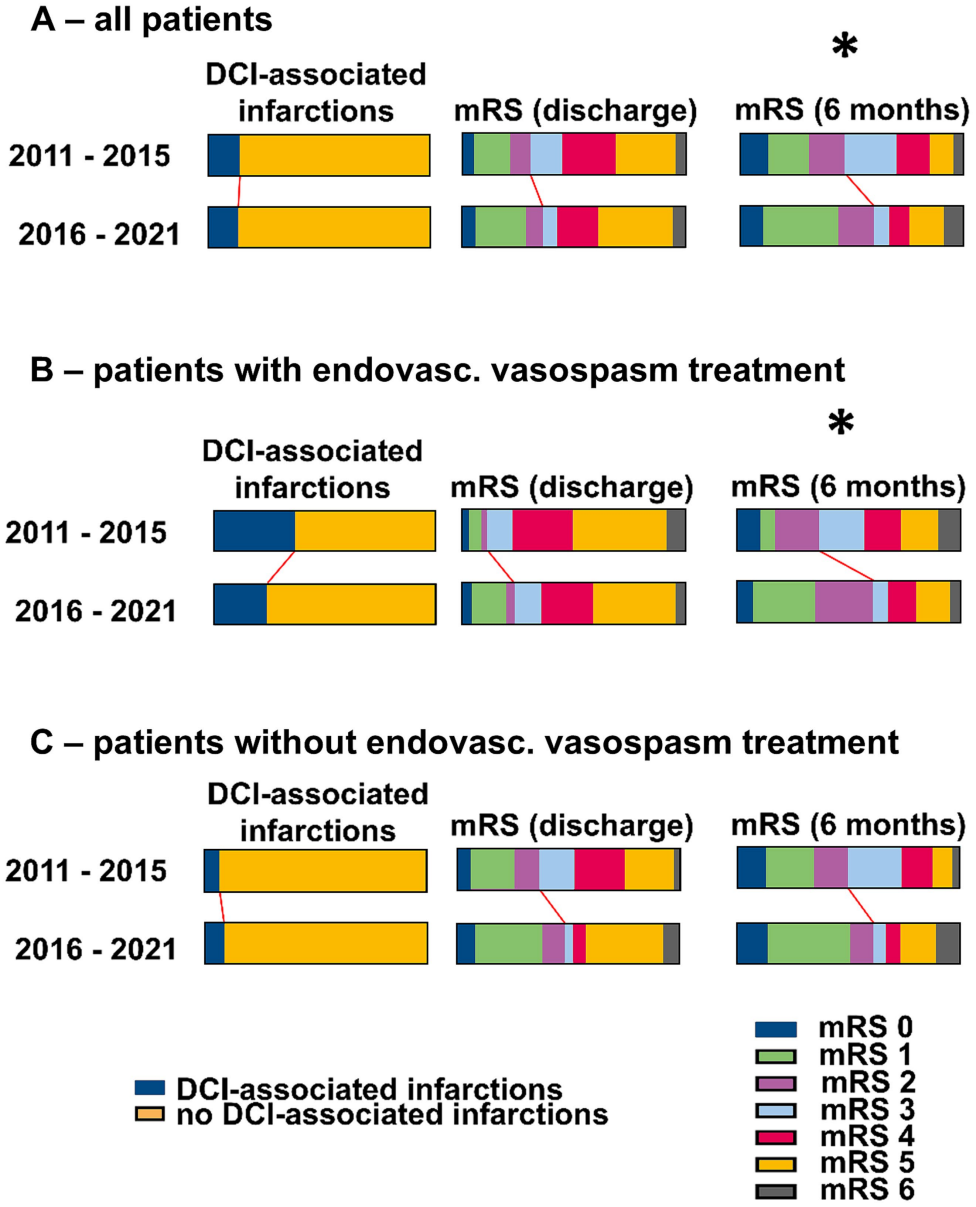
Outcomes in the cohorts before and after the introduction of ciaN. **(A)** All patients. **(B)** Patients with endovascular vasospasm treatments. **(C)** Patients without endovascular vasospasm treatments. *p < 0.05 (Fisher’s exact test).

Overall, among the 292 included patients, 39 DCI-associated cerebral infarctions (13.4%) were noted. Overall, no significant differences in the rates of DCI-associated infarctions were observed between the pre-ciaN and ciaN groups (13.8% vs. 12.9%, *n.s.*). Patients who received endovascular vasospasm treatments after the introduction of ciaN tended to experience less DCI-associated infarctions (pre-ciaN vs. ciaN: 36.1% vs. 23.5%, *n.s.*). Figure 3 summarizes the data on the occurrence of DCI-associated infarctions in more detail.

**Figure 4:**
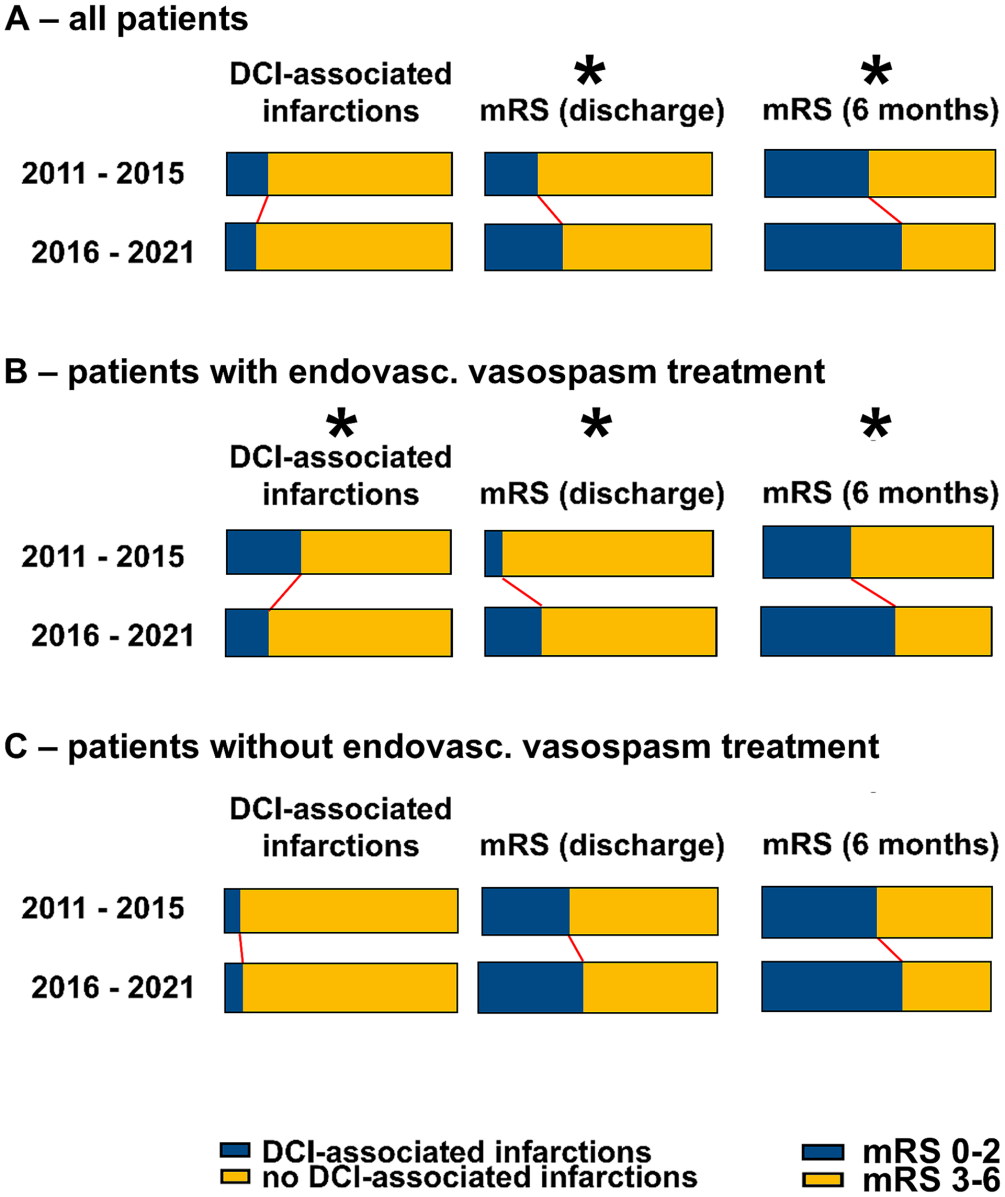
Weighted pseudopopulation—outcomes in the cohorts before and after the introduction of ciaN. The Figure shows the results from evaluation of the weighted pseudopopulation after propensity score matching. **(A)** All patients. **(B)** Patients with endovascular vasospasm treatments. **(C)** Patients without endovascular vasospasm treatments. *p < 0.05 (Fisher’s exact test).

After propensity score matching, there were still no significant differences in the rates of DCI-associated infarctions between the pre-ciaN and ciaN groups. However, when considering the subgroups of patients who received endovascular vasospasm treatment, there were significantly less DCI-associated infarctions in the ciaN group (18.9% vs. 33.0%, *p* < 0.05), while there was no significant difference for those patients who did not receive endovascular vasospasm treatments. Figure 4 summarizes the data on the occurrence of DCI-associated infarctions in the weighted pseudo-sample after propensity score matching in more detail.

### Clinical outcome

Overall, of the 292 included patients, 98 (33.6%) were discharged with mRS scores of 0–2, and 194 (66.4%) were discharged with mRS scores of 3–6. There was a higher rate of patients discharged with favorable mRS scores in the ciaN group, which did not reach statistical significance (mRS 0–2, pre-ciaN vs. ciaN groups: 30.3% vs. 36.7%, *n.s.*). This was more pronounced in the subgroup of patients who received endovascular vasospasm treatments (mRS 0–2, endovascular treatment, pre-ciaN vs. ciaN groups: 11.1% vs. 23.5%, *n.s.*; mRS 0–2, *no* endovascular treatment, pre-ciaN vs. ciaN groups: 34.9% vs. 43.8%, *n.s.*).

Six-month follow-up data were available for 244 patients (83.6%, pre-ciaN cohort: 84.1%, ciaN cohort: 83.0%), of whom 131 (53.7%) achieved favorable outcomes with mRS scores of 0–2. There were significantly more patients with favorable outcomes at 6 months in the ciaN group (mRS 0–2, pre-ciaN vs. ciaN groups: 46.7*% vs.* 60.7%, p < 0.05). This difference between the pre-ciaN and ciaN groups was most pronounced in patients who received endovascular vasospasm treatments (mRS 0–2, endovascular treatment, pre-ciaN vs. ciaN groups: 36.7% vs. 63.0%, p < 0.05; mRS 0–2, *no* endovascular treatment, pre-ciaN vs. ciaN groups: 50.0% vs. 60.5%, *n.s.*), and in patients with H&H scores 1–3 (mRS 0–2, H&H 1–3, pre-ciaN vs. post-ciaN: 56.0% vs. 78.2%, p < 0.01). Figure 3 summarizes neurological outcomes in more detail.

After propensity score matching, outcomes at discharge and at 6 months were significantly better in the ciaN group (at discharge, mRS 0–2, pre-ciaN *vs.* ciaN groups: 23.2% vs. 34.0%, p < 0.05; at 6 months, mRS 0–2, pre-ciaN vs. ciaN groups: 44.8% vs. 59.6%, p < 0.01). When considering the subgroups of patients who received endovascular vasospasm treatment, the difference was markedly larger (endovasc. Vasospasm therapy, at discharge, mRS 0–2, pre-*ciaN vs. ciaN* groups: 7.2% vs. 24.2%, p < 0.05; at 6 months, mRS 0–2, pre-ciaN vs. ciaN groups: 38.0% vs. 58.1%, p < 0.01). Conversely, in the subgroup of patients who did not receive endovascular vasospasm therapies, there were no statistical differences in outcome at discharge or at 6 months. This indicates a contribution of the change in treatment standard for endovascular vasospasm therapies from angioplasties to ciaN.

## Discussion

In our clinical center, the standard of endovascular vasospasm therapy for patients with SAH and refractory DCI was changed in 2016. In previous years, angioplasty of vasospastic vessel segments was performed in cases of DCI refractory to induced hypertension. Since 2016, angioplasty has been performed only in exceptional cases, whereas ciaN has been performed as the standard endovascular vasospasm therapy. For ciaN, an intra-arterial catheter is inserted into the internal carotid or VA, and nimodipine is continuously infused over several days. Antiplatelet therapy with tirofiban or anticoagulation is necessary during ciaN to prevent thromboembolic complications. In this study, patients treated after SAH in our center were retrospectively recorded over 10 years. The outcomes in the years before and in the years after the change in standard endovascular vasospasm therapy were then compared.

The most important finding was that the clinical outcomes at 6 months after SAH significantly improved after switching to ciaN. After propensity score matching, there was a significantly improved outcome at discharge and after 6 months as well as a reduced rate of DCI-associated infarctions in the ciaN cohort only in the subgroup of patients with endovascular vasospasm treatment. This indicates a contribution of the changes in treatment standard from angioplasties to ciaN to the improved outcomes.

Interestingly, we observed that in patients who were admitted with H&H scores of 1–3, 6-month outcomes improved by a markedly smaller, however, significant extent since 2016 also in patients who were not treated endovascularly for vasospasms (Supplementary tables), indicating that other factors may have also contributed to the improved outcomes. The improvement is most likely due to general improvements, particularly of endovascular and surgical aneurysm occlusion and intensive care therapy (22). The detailed cause cannot be determined by the current evaluation approach.

During angioplasty, a balloon is inserted into the vasospastic vascular segment and expanded under a defined pressure (23, 24). This approach results in the elimination of vasospasm. The structure of the vessel wall is initially impaired by angioplasty, so that subsequent vasospasm does not immediately reoccur in the angioplasty segment. During ciaN, a microcatheter is inserted into the ICA or VA. This is used to continuously infuse nimodipine into the vasospastic vascular bed. Therefore, not only the vasospasm of the large vessels but also microvasospasms are targeted, which play an important role (5). In line with this pathophysiological concept, our group has shown that by infusing nimodipine directly into the vasopastic vascular bed, a reduction in vasospasm of the large vessels and a significant increase in cerebral perfusion can be achieved (25). The better outcomes in patients treated with ciaN could therefore be explained by the fact that ciaN not only reduces angiographic vasospasm of the large cerebral vessels but also has an effect on the small vessels, which is crucial for cerebral perfusion. We and others have further reported that ciaN is a safe procedure and that complications are rare (9–14).

In a prospective, randomized study by Vatter et al. (26), the effects of endovascular vasospasm therapy (angioplasty or single-stage intra-arterial pharmacological vasospasmolysis) were compared with those of the best medical management (induced hypertension). The authors found that endovascular therapy resulted in worse neurological outcomes. In our study, no statement can be made about the effects of the best medical management because all patients with refractory DCI were offered endovascular treatment. It is therefore not possible to make a statement regarding whether ciaN is superior to the best medical management. We cannot rule out that the observed improvement in outcomes is solely due to the avoidance of angioplasty and that ciaN is not better than the best medical management. In our opinion, a prospective clinical study comparing ciaN with the best medical management for treating DCI after SAH refractory to induced hypertension is warranted.

The neurological outcomes significantly improved after the introduction of ciaN. In contrast, no significant reduction in the number of DCI-associated infarctions was observed. In both groups, before and after the introduction of ciaN, DCI-associated infarctions were in most cases not territorial infarctions, but rather small subterritorial lesions, with presumably often a lack of functional relevance. This implies that the observed improvement in outcomes is not because of the prevention of infarctions, but rather due to the neuroprotective effects of ciaN through improved cerebral perfusion compared with angioplasty. Interestingly, a study by Vatter et al. (26) also reported no difference in the frequency of DCI-associated infarctions, despite a significant difference in neurological outcomes.

## Conclusion

Our analysis revealed that switching from angioplasty to continuous intra-arterial vasospasmolysis as the standard endovascular treatment for refractory DCI after SAH significantly improved clinical outcomes. It remains unclear whether the improvement in outcomes is due only to the avoidance of angioplasty or whether ciaN also improves outcomes compared with the best medical management. A prospective clinical study comparing ciaN with the best medical management for treating DCI after SAH refractory to induced hypertension is therefore warranted.

## Limitations

Our study is a retrospective single-center evaluation. The retrospective character can cause selection and information bias. Furthermore, it limits the generalizability of the results. Additionally, there were heterogeneities in the patient cohorts; propensity score matching was used, however, it is not possible to control for all relevant confounders.

## Data Availability

The original contributions presented in the study are included in the article/Supplementary material, further inquiries can be directed to the corresponding authors.

## Footnotes

^1^ https://www.graphpad.com/quickcalcs/contingency1/
