# Supplementary material for: Clinical outcomes in spontaneous subarachnoid hemorrhage after introduction of continuous intra-arterial vasospasmolysis for treatment of refractory delayed cerebral ischemia
Source: Front Neurol. 2025 Dec 3;16:1636083. doi: 10.3389/fneur.2025.1636083 (PMC12709924; doi:10.3389/fneur.2025.1636083)
Supplement: Supplementary file 1 [file Table_1.DOCX]

|  |  | 2011-2015 | 2016-2020 |  |
| --- | --- | --- | --- | --- |
|  |  |  |  |  |
| all patients | DCI | 20 (13.8 %) | 19 (12.9 %) | *n.s.* |
|  | no DCI | 125 (86.2 %) | 128 (87.1 %) |  |
|  |  |  |  |  |
| H&H 1-3 | DCI | 10 (9.4 %) | 9 (9.6 %) | *n.s.* |
|  | no DCI | 96 (90.6 %) | 85 (90.4 %) |  |
|  |  |  |  |  |
| H&H 4-5 | DCI | 10 (25.6 %) | 10 (18.9 %) | *n.s.* |
|  | no DCI | 29 (74.4 %) | 43 (81.1 %) |  |
|  |  |  |  |  |
| all patients, ET | DCI | 13 (36.1 %) | 12 (23.5 %) | *n.s.* |
|  | no DCI | 23 (63.9 %) | 39 (76.5 %) |  |
|  |  |  |  |  |
| H&H 1-3, ET | DCI | 7 (29.2 %) | 6 (18.8 %) | *n.s.* |
|  | no DCI | 17 (70.8 %) | 26 (81.2 %) |  |
|  |  |  |  |  |
| H&H 4-5, ET | DCI | 6 (50 %) | 6 (31.6 %) | *n.s.* |
|  | no DCI | 6 (50 %) | 13 (68.4 %) |  |
|  |  |  |  |  |
| all patients, no ET | DCI | 7 (6.4 %) | 7 (7.3 %) | *n.s.* |
|  | no DCI | 102 (93.6 %) | 89 (92.7 %) |  |
|  |  |  |  |  |
| H&H 1-3, no ET | DCI | 3 (3.7 %) | 3 (4.8 %) | *n.s.* |
|  | no DCI | 79 (96.3 %) | 59 (95.2 %) |  |
|  |  |  |  |  |
| H&H 4-5, no ET | DCI | 4 (14.8 %) | 4 (11.8 %) | *n.s.* |
|  | no DCI | 23 (85.2 %) | 30 (88.2 %) |  |

**Table 1 A: DCI-associated infarctions**

DCI indicates DCI-associated infarctions; ET – endovascular treatment

|  |  | 2011-2015 | 2016-2020 |  |
| --- | --- | --- | --- | --- |
|  |  |  |  |  |
| all patients | mRS 0-2 | 44 (30.3 %) | 54 (36.7 %) | *n.s.* |
|  | mRS 3-6 | 101 (69.7 %) | 93 (63.3 %) |  |
|  |  |  |  |  |
| H&H 1-3 | mRS 0-2 | 42 (39.6 %) | 46 (48.9 %) | *n.s.* |
|  | mRS 3-6 | 64 (60.4 %) | 48 (51.1 %) |  |
|  |  |  |  |  |
| H&H 4-5 | mRS 0-2 | 2 (5.1 %) | 9 (17.0 %) | *n.s.* |
|  | mRS 3-6 | 37 (94.9 %) | 44 (83.0 %) |  |
|  |  |  |  |  |
| all patients, ET | mRS 0-2 | 4 (11.1 %) | 12 (23.5 %) | *n.s.* |
|  | mRS 3-6 | 32 (88.9 %) | 39 (76.5 %) |  |
|  |  |  |  |  |
| H&H 1-3, ET | mRS 0-2 | 4 (16.7 %) | 10 (31.3 %) | *n.s.* |
|  | mRS 3-6 | 20 (83.3 %) | 22 (68.8 %) |  |
|  |  |  |  |  |
| H&H 4-5, ET | mRS 0-2 | 0 (0.0 %) | 2 (10.5 %) | *n.s.* |
|  | mRS 3-6 | 12 (100.0 %) | 17 (89.5 %) |  |
|  |  |  |  |  |
| all patients, no ET | mRS 0-2 | 38 (34.9 %) | 42 (43.8 %) | *n.s.* |
|  | mRS 3-6 | 71 (65.1 %) | 54 (56.2 %) |  |
|  |  |  |  |  |
| H&H 1-3, no ET | mRS 0-2 | 38 (46.3 %) | 36 (58.1 %) | *n.s.* |
|  | mRS 3-6 | 44 (53.7 %) | 26 (41.9 %) |  |
|  |  |  |  |  |
| H&H 4-5, no ET | mRS 0-2 | 2 (7.4 %) | 6 (17.6 %) | *n.s.* |
|  | mRS 3-6 | 25 (92.6 %) | 28 (82.4 %) |  |

**Table 1 B: Outcome at hospital discharge**

ET indicates endovascular vasospasm treatment; ET – endovascular treatment

|  |  | 2011-2015 | 2016-2020 |  |
| --- | --- | --- | --- | --- |
|  |  |  |  |  |
| all patients | mRS 0-2 | 57 (46.7 %) | 74 (60.7 %) | *** |
|  | mRS 3-6 | 65 (53.3 %) | 48 (39.3 %) | *p = 0.0397* |
|  |  |  |  |  |
| H&H 1-3 | mRS 0-2 | 51 (56.0 %) | 61 (78.2 %) | *** |
|  | mRS 3-6 | 40 (44.0 %) | 17 (21.8 %) | *p = 0.0032* |
|  |  |  |  |  |
| H&H 4-5 | mRS 0-2 | 6 (19.4 %) | 13 (29.5 %) | *n.s.* |
|  | mRS 3-6 | 25 (80.6 %) | 31 (70.5 %) |  |
|  |  |  |  |  |
| all patients, ET | mRS 0-2 | 11 (36.7 %) | 29 (63.0 %) | *** |
|  | mRS 3-6 | 19 (63.3 %) | 17 (37.0 %) | *p = 0.0345* |
|  |  |  |  |  |
| H&H 1-3, ET | mRS 0-2 | 8 (40.0 %) | 21 (77.8 %) | *** |
|  | mRS 3-6 | 12 (60.0 %) | 6 (22.2 %) | *p = 0.0146* |
|  |  |  |  |  |
| H&H 4-5, ET | mRS 0-2 | 3 (30.0 %) | 3 (15.8 %) | *n.s.* |
|  | mRS 3-6 | 7 (70.0 %) | 12 (63.2 %) |  |
|  |  |  |  |  |
| all patients, no ET | mRS 0-2 | 46 (50.0 %) | 46 (60.5 %) | *n.s.* |
|  | mRS 3-6 | 46 (50.0 %) | 30 (39.5 %) |  |
|  |  |  |  |  |
| H&H 1-3, no ET | mRS 0-2 | 43 (60.6 %) | 40 (78.4 %) | *** |
|  | mRS 3-6 | 28 (39.4 %) | 11 (21.6 %) | *p = 0.0489* |
|  |  |  |  |  |
| H&H 4-5, no ET | mRS 0-2 | 3 (14.3 %) | 6 (24.0 %) | *n.s.* |
|  | mRS 3-6 | 18 (85.7 %) | 19 (76.0 %) |  |

**Table 1 C: Outcome at 6 months**

ET indicates endovascular vasospasm treatment; ET – endovascular treatment

* p < 0.05
